# A Novel Implant Design for Cemented Endoprosthesis Stems to Reduce the Risk of Bone Cement Implantation Syndrome

**DOI:** 10.7759/cureus.76918

**Published:** 2025-01-04

**Authors:** Ugo N Udogwu, Jessa D Fogel, Danielle Sim, Mario N Sahlani, Ryan Smith, Kevin R Aroom, Vincent Y Ng

**Affiliations:** 1 Department of Orthopedics, University of Maryland School of Medicine, Baltimore, USA; 2 Robert E. Fischell Institute for Biomedical Devices, University of Maryland, Baltimore, USA

**Keywords:** arthroplasty, biomechanics, bone cement implantation syndrome, femur stem, implant design, reconstruction

## Abstract

Background: Bone cement implantation syndrome (BCIS) is a life-threatening risk of cemented stems. There are limited methods to prevent BCIS and few published studies. A survey of the Musculoskeletal Tumor Society (MSTS) surgeons was conducted to evaluate their experiences with BCIS. A novel stem designed to reduce intramedullary pressure during insertion was evaluated.

Methods: A survey was distributed to MSTS members, and data was collected. The novel stem featured a hollow longitudinal channel, an entry hole at the tip, and an egress hole at the collar for cement to flow from the intramedullary canal during insertion. Bending stiffness was compared using finite element analysis to a standard solid stem. Stems were cemented into cadaveric femurs. Specimens were loaded with 8000 N tensile force and then maximally torqued until failure. Intramedullary pressures were measured for novel and standard stems cemented into sawbones.

Results: In 107 survey responses, 58% (n = 63) experienced severe BCIS, and 83% (n = 52) of those had ≥1 death from BCIS complications. Many surgeons avoid cementing long stems, and 78% (n = 14) report concern for BCIS as the reason. Seventy-nine percent (n = 84) use an average of 4.75 different methods to reduce BCIS risk. The novel stem demonstrated 2.8% reduced bending stiffness. When cemented into cadaveric bone, both stem designs achieved 8000 N of tensile force, and there was no significant difference in torque failure (140.6 Nm in the novel stem; 128 Nm in the standard stem). The average peak pressure was significantly lower for the novel stem (77 psi vs. 151 psi).

Conclusions: The majority of surgeons have concerns regarding BCIS based on their experience and use multiple methods to reduce risk. A novel stem can reduce the intramedullary insertion pressure by approximately half and possibly reduce the risk of BCIS. Testing demonstrates similar stiffness and stability compared to standard solid stems.

## Introduction

Bone cement is a reliable method of securing orthopedic prosthetic stems. Bone cement implantation syndrome (BCIS) is a well-described phenomenon and potential sequela of this implantation technique. When a solid stem is inserted into the canal filled with cement, there is a significant increase in intramedullary pressure. This causes embolization of fat, air, cement particles, and aggregates of platelets, fibrin, and bone marrow into the venous system, which subsequently causes right heart dysfunction, increases pulmonary circulation pressures, and can lead to cardiovascular collapse [1,2]. Various studies have shown the incidence of all types of BCIS to be 25%-55% of patients undergoing cemented arthroplasty [3-7]. The incidence further increases to about 75% in patients with cancer undergoing orthopedic procedures [8]. The mortality rate from BCIS has been reported to be 0.2%-1.6% in elderly patients with hip fractures and up to 4.3% in patients with cancer-related hip fractures [9]. Although there are surgical and anesthetic measures to attenuate the risk, none are completely effective, and BCIS remains a significant risk for intraoperative morbidity and, occasionally, mortality. Previous research has focused predominantly on how to mitigate this complication when it occurs as opposed to preventative measures [10-14].

The purposes of this study were (1) to assess the level of experience and amount of concern among musculoskeletal tumor surgeons regarding BCIS, (2) to evaluate the biomechanical properties of a novel stem design compared to a standard stem design, and (3) to measure the intramedullary insertion pressures during cementation of a novel stem design compared to a standard stem design. 

The novel stem examined in this study features a channel longitudinally within the stem to allow cement to flow through the stem during implantation. There is an egress hole to the channel at the collar to allow extruded cement to flow out. Unlike solid stems which act similarly to the “plunger” of a syringe when inserted into a closed intramedullary canal filled with cement and can generate high intramedullary pressures, the novel stem is designed to mitigate this risk. Minimally acceptable pressures of 14.5 psi (100 kPa) are needed to produce adequate cement-bone interdigitation and implant stability [15]. Actual applied pressures can vary from 50 to 218 psi (350-1500 kPa) [16]. As such, a stem designed to reduce cementation pressure could still achieve minimal acceptable pressure for stability. The novel stem has longitudinal scallops on the outer aspect of the stem similar to standard stems. 

## Materials and methods

Methods

Table 1 shows the BCIS grading system used in this study.

**Table 1:** Bone cement implantation syndrome (BCIS) grading CPR: cardiopulmonary resuscitation Source: [2]

Severity	Criteria (must meet at least 1)
Grade 1	Moderate hypoxia (SpO2 < 94%); fall in systolic blood pressure > 20%
Grade 2	Severe hypoxia (SpO2 < 88%); fall in systolic blood pressure > 40%; unexpected loss of consciousness
Grade 3	Cardiovascular collapse requiring CPR

Survey Distribution

A survey requesting demographic information and experience with BCIS was created on REDCap (Vanderbilt University, Nashville, TN, USA). After receiving permission from the Musculoskeletal Tumor Society (MSTS) Membership and Executive Committees, the survey was distributed to all MSTS members. Responses were accepted for one month after distribution. A follow-up email was sent one week before the deadline to encourage more participation. The survey included 11 questions about respondent demographics, tumor practice information, experience with BCIS, and use of prevention strategies (Appendix A). Descriptive statistics were used to characterize respondent demographics, practice information, experience with BCIS, and use of preventative techniques. Chi-squared and Fisher’s exact tests were used to determine associations between categorical variables.

Biomechanical Testing of Stems

**Figure 1:**
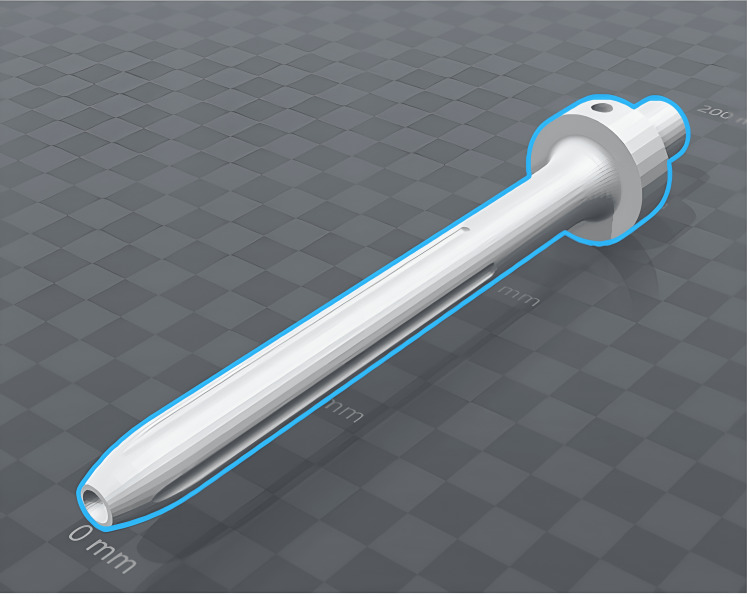
Computer-aided design (CAD) prototype of a novel stem Computer-aided design (CAD) prototype of a novel stem with an opening at the tip of the stem to an inner longitudinal channel. There is an egress hole at the collar for extruded cement. Trunnion for the megaprosthesis body sits proximal to the collar Image credit: Vincent Y. Ng, MD; Ryan Smith

**Figure 2:**
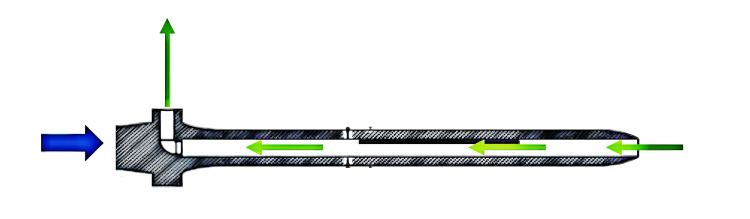
Internal design of the novel stem As the stem is inserted (blue arrow), some of the cement within the intramedullary canal of the bone can flow (green arrow) into the channel within the stem and out of the egress hole in the collar Image credit: Vincent Y. Ng, MD; Ryan Smith

A 13 mm diameter cylindrical straight stem with a 5.5 mm egress channel was designed for the diaphysis of the bone (Figures 1-2). The surface of the stem featured a scalloped design to facilitate stability to torsional stress in the cement mantle similar to the outer contour of currently available cemented stems for megaprostheses [17]. A finite element analysis was performed to compare the stiffness of the proposed hollow prototype stem to that of the current solid stem technology. Five standard and five novel stems were designed using a computer-aided design (CAD) program (SolidWorks, Inc., Waltham, Massachusetts, USA). The only difference between the two types of stems was the central longitudinal channel with the egress hole at the collar for the novel stems. The CAD plans were sent to a third party (Materialise, Leuven, Belgium) to be 3D printed in stainless steel. 

Ten fresh-frozen femora (five matched pairs) with a mean age of 68.2 +/- 7.8 years (range 57 to 76 years) were obtained from a local cadaver donor organization. The femora were obtained pre-thawed with all soft tissue removed. Of the five cadaveric donors, two were male and three were female donors. The specimens were excluded for the following reasons: younger than 50 years, history of prior surgery on femora, history of tumors and/or metastatic disease, infection, or metabolic syndrome, and history of prior femoral fracture.

A proximal femur osteotomy was performed 5 cm below the distal margin of the lesser tuberosity to accommodate the implant. A second distal osteotomy was made 12 cm proximal to the joint to produce the final diaphyseal testing specimen for each of the 10 femora. Each specimen was reamed to 15 mm for a 2 mm cement mantle. For each matched pair, one femur was implanted with a standard stem, and the contralateral femur was implanted with the novel stem. Standard third-generation cementation technique was utilized during implantation and included cement plug/restrictor, lavage, vacuum mixing, retrograde filling of the canal with a cement gun, and pressurization. The stems were implanted using polymethylmethacrylate (PMMA) cement (Simplex P Radiopaque Bone Cement, Stryker, Mahwah, NJ) without antibiotics, which was mixed for 1.5 minutes prior to prompt filling and implantation.

**Figure 3:**
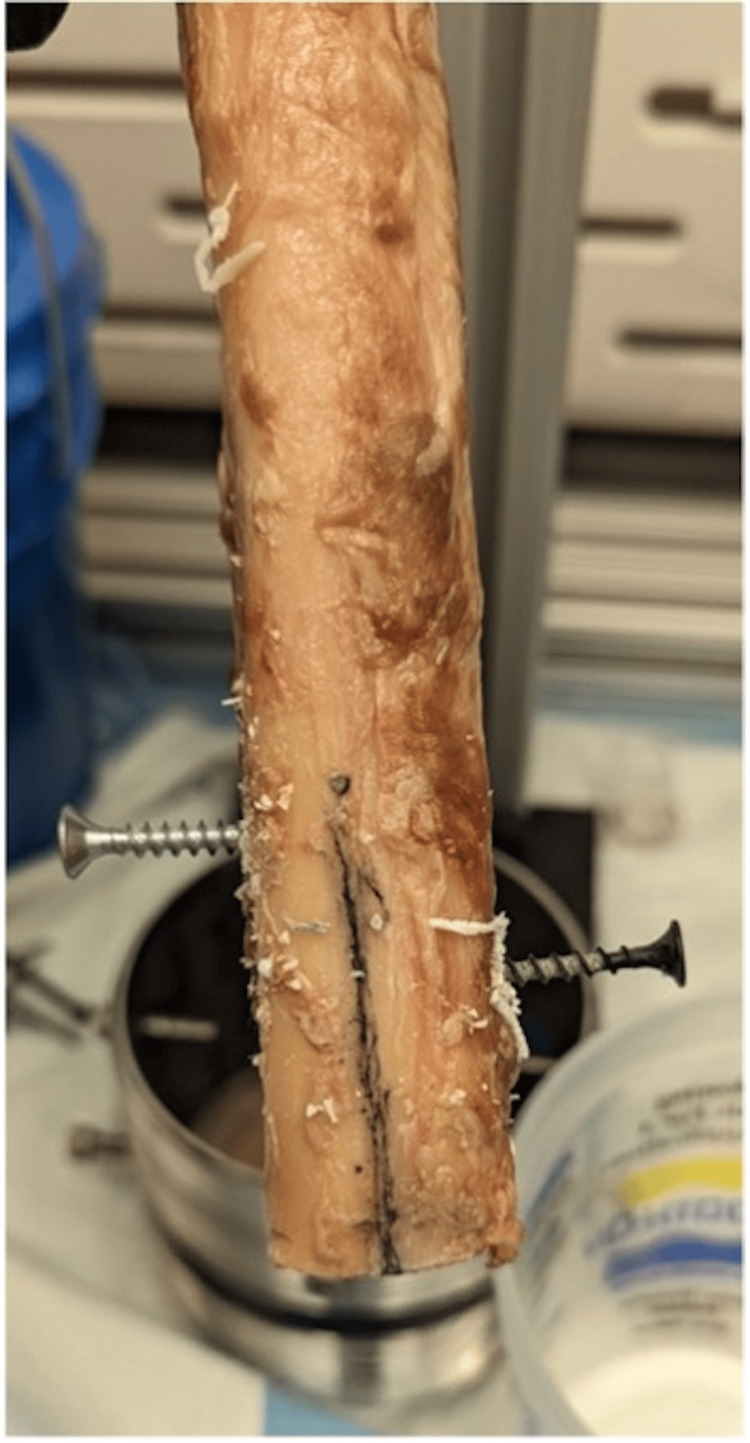
Structural screws driven into the distal aspect of the implanted specimen to improve mechanical locking of the bone into the potting resin Image credit: Vincent Y. Ng, MD; Kevin R. Aroom, MS

**Figure 4:**
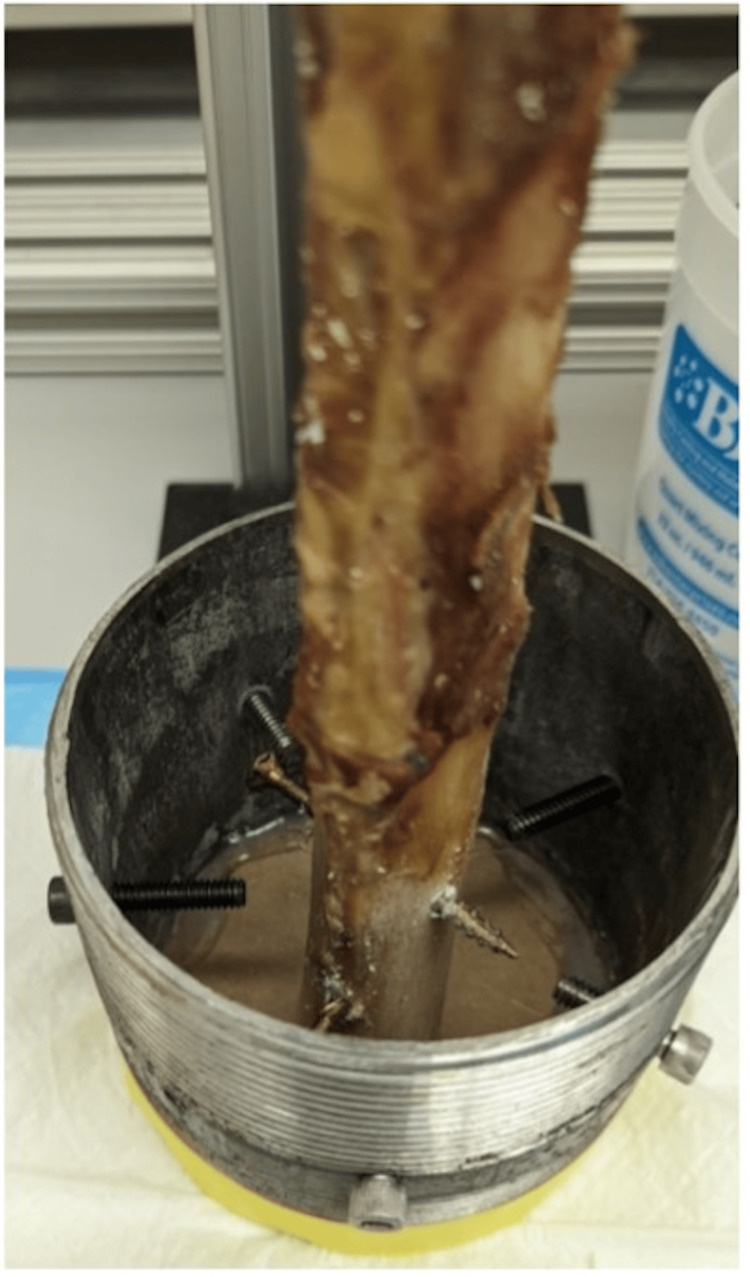
Implanted specimen in a 4-inch iron pipe fitting in preparation for urethane casting resin Image credit: Vincent Y. Ng, MD; Kevin R. Aroom, MS

**Figure 5:**
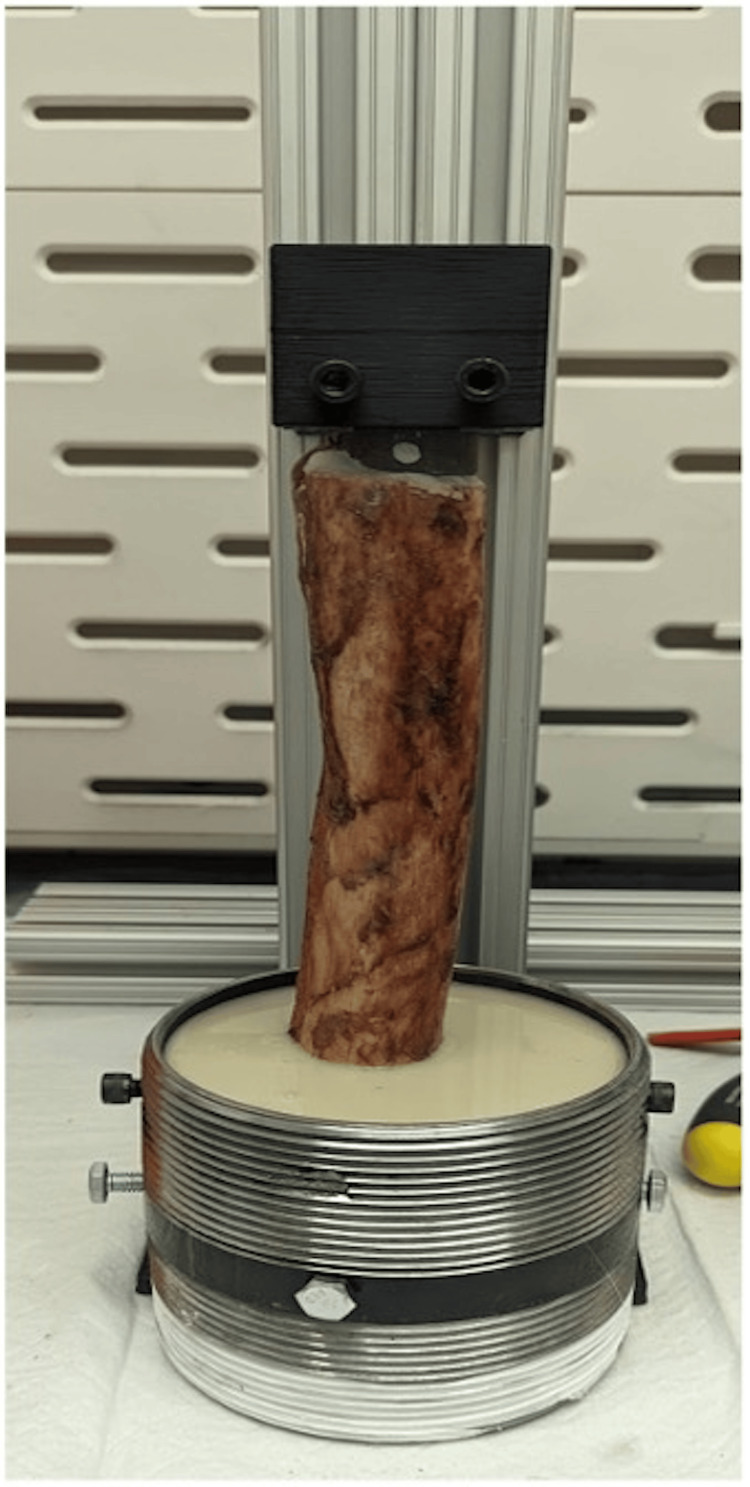
Final potting construct after resin curing for at least 60 minutes Image credit: Vincent Y. Ng, MD; Kevin R. Aroom, MS

Following the implantation of the stems, each specimen was potted to facilitate biomechanical testing. Each specimen had two pilot holes drilled perpendicular to the major axis of the bone, with #10 2.5-inch structural screws driven in to improve the mechanical locking of the bone to the potting resin. The distal ends of the femora were potted in a 4-inch national pipe tapered thread iron pipe nipple fitting using a urethane casting resin (SmoothCast 380, Smooth-On), with the resin curing for at least 60 minutes (Figures 3-5). Tensile loading was applied using a universal testing system (Tinius Olsen H25K-T) at a rate of approximately 0.02 mm/sec to a proof tensile load of 8000 N. A Tinius Olson 602 Torsion Testing System was used to apply 4 degrees per minute of torsional displacement until bone fracture, implant failure, or 200 Nm of torsion was reached. A max torsion of 200 Nm was chosen based on prior literature demonstrating standardized loads acting on hip implants [18]. Peak torque prior to fracture or implant failure was recorded.

Implantation Pressure Testing

Stems of 13 mm diameter were 3D printed (five standard, five novel) with carbon-fiber infused nylon filament. The novel stems had a 5.5 mm longitudinal channel and egress hole. In order to standardize the testing conditions, bone replicas (“Sawbones”) were used instead of cadaveric bone. The Sawbones (Pacific Research Laboratories, Inc., Vashon, WA, USA) were customized for this study using fourth-generation cylindrical models with 35 mm outer diameter, 7 mm cortical wall, and #15 foam for cancellous bone. The Sawbone was cut to a length 3 cm longer than the implanted portion of the stem. In order to measure intramedullary pressure, a manometer (Measureman Pressure Gauge, Paris, France) was attached to the Sawbone using a PVC pipe adapter which was sealed with PVC primer and cement (Oatey Co, Cleveland, OH, USA). A small amount of petroleum jelly (Vaseline, Unilever Co., London, England) was placed over the diaphragm of the manometer in order to transmit pressure from the PMMA cement and to prevent intrusion of the PMMA cement into the manometer itself. The manometer was situated at the end of the cemented canal because although there is no widely accepted or published method of measuring intramedullary pressures during cementation, a prior study indicated that the intramedullary pressures generated are typically highest near the distal end of the cemented canal [16].

**Figure 6:**
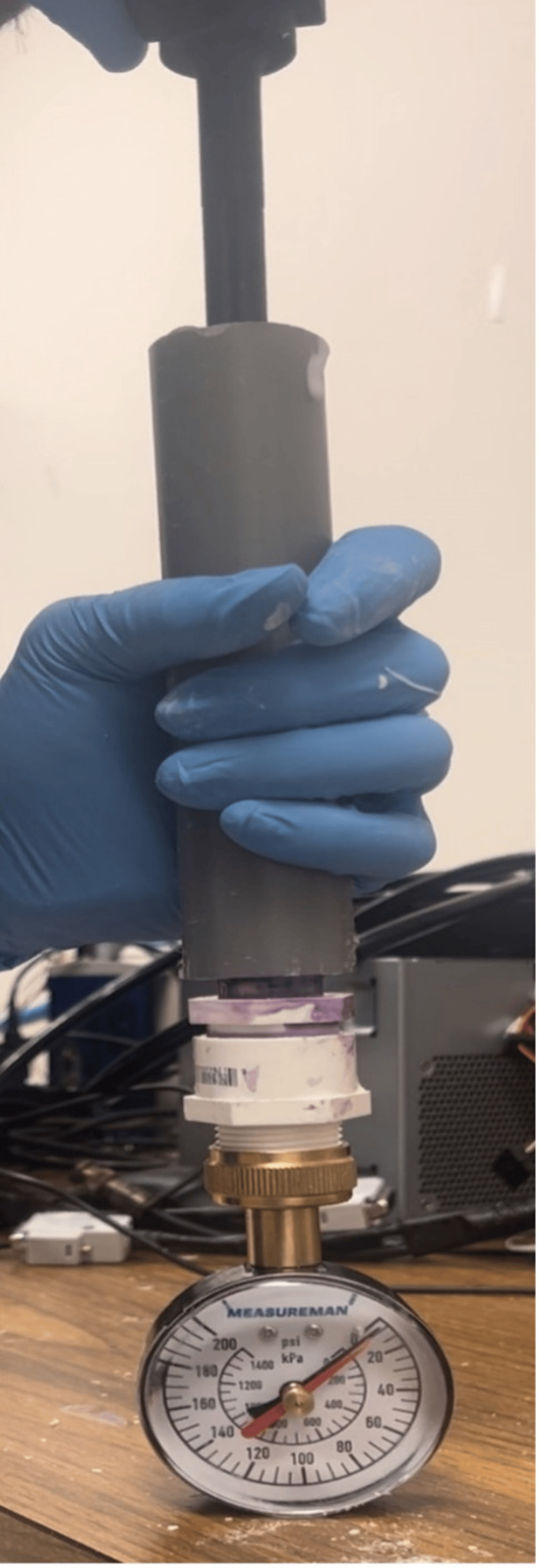
Experimental setup to measure intramedullary pressure during cementation Image credit: Vincent Y. Ng, MD

Standard third-generation cementation technique was utilized during implantation and included pressurization, vacuum mixing, and retrograde filling of the canal with a cement gun. The stems were implanted using PMMA cement (Simplex P Radiopaque Bone Cement, Stryker) without antibiotics, which was mixed for 1.5 minutes prior to prompt filling and implantation. The maximal resulting pressure during insertion of the stem was recorded (Figure 6).

## Results

Results

Survey Results

**Figure 7:**
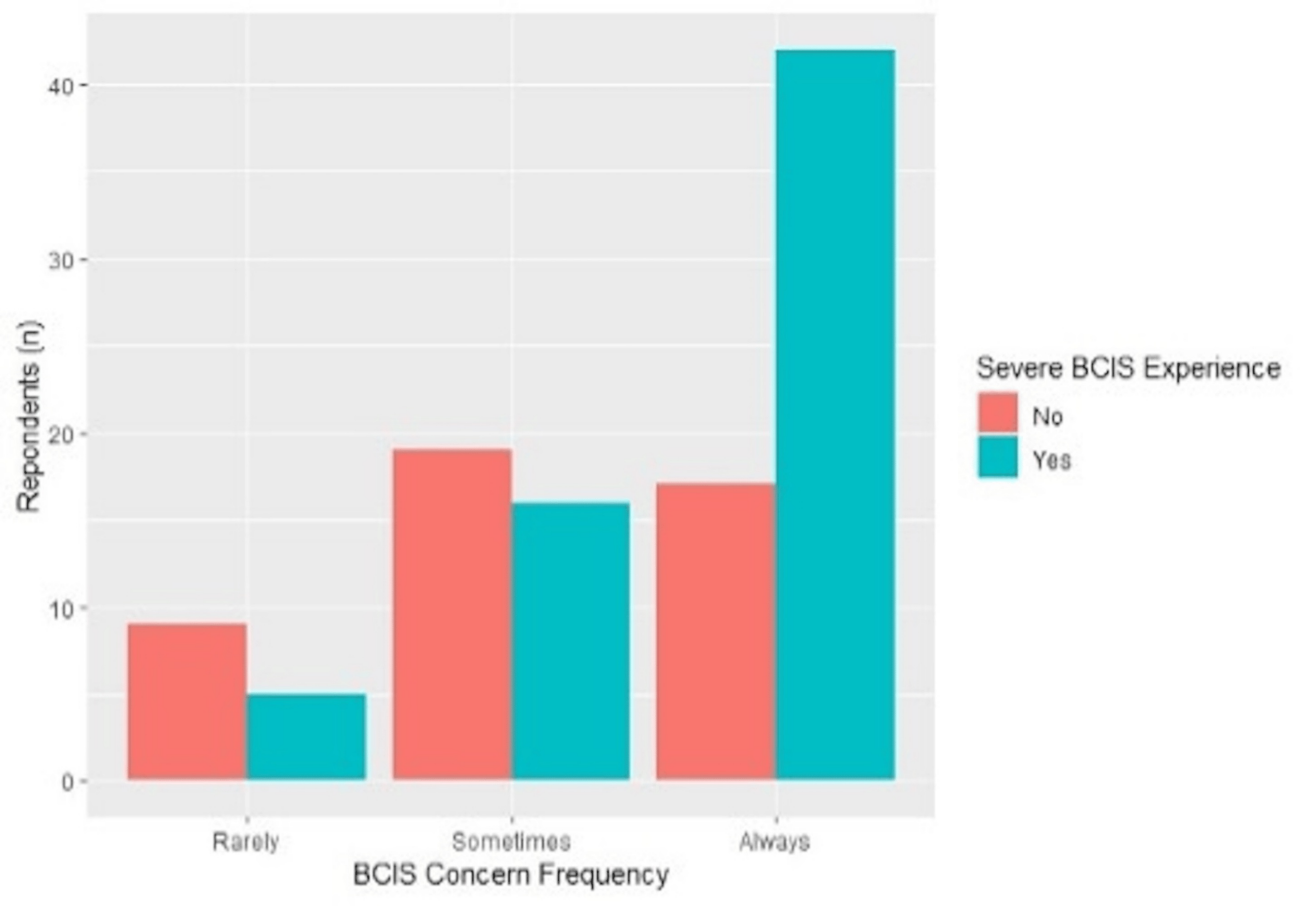
BCIS concern among respondents based on the incidence of severe BCIS BCIS: bone cement implantation syndrome

A total of 107 responses were received. A total of 58% (n = 63) of all respondents had experienced severe BCIS (grade 3) during their practice. A total of 70% (n = 16) of surgeons who had been in practice for 15-20 years reported patients who experienced severe BCIS (Table 2). Of the respondents who had experienced severe BCIS, 82.5% (n = 52) reported at least one patient death from intraoperative or postoperative complications. Those who had experienced severe BCIS were significantly more likely to report always having concern when implanting cemented stems compared to those who did not (66% vs. 37%, p = 0.005) (Figure 7). 

**Table 2:** Grade 3 BCIS experience among respondents IQR: interquartile ranges; SD: standard deviation

Years in practice	# of respondents (n)	BCIS grade 3 experience n (%)	Median (IQR) patients with BCIS grade 3	Mean number of deaths associated with BCIS grade 3 n (SD)
0-5	18	10 (56)	2 (1-2)	1 (1-1)
5-10	49	29 (59)	2 (1-3)	1 (1-2)
10-15	18	8 (44)	2 (2-4)	1 (0.75-2.3)
15-20	23	16 (70)	4 (3-5)	2 (0.50-2)
Overall	107	63 (58)	2 (2-4)	1 (1-2)

For elderly (>75 years old) patients, respondents on average used cemented stems 86.9% of the time (SD = 23.9%). Seventy-two percent (n = 78) of surgeons reported using long cemented stems, and 16% (n = 18) did not. For those that use long cemented stems, the use of long cemented stems occurred with a median (IQR) of 15% (5-25) of the time. For those who did not use long-cemented stems, 77.8% (n = 14) of respondents cited concern for BCIS as the reason they did not.

For surgeons’ estimated risk of BCIS, 84% (n = 90) classified cancer patients with American Society of Anesthesiologists (ASA) grade 3 or greater as “Intermediate” or “High” risk for BCIS grade 1. For BCIS grade 3, 23% (n = 25) of respondents considered these patients at “Intermediate” or “High” risk. When considering elderly (>75 years old) cancer patients, 77% (n = 82) of respondents rated the risk of BCIS grade 1 as “Intermediate” or “High” and 7.5% (n = 7) classified the risk of BCIS grade 3 in these patients as “Intermediate” or “High.” 

**Figure 8:**
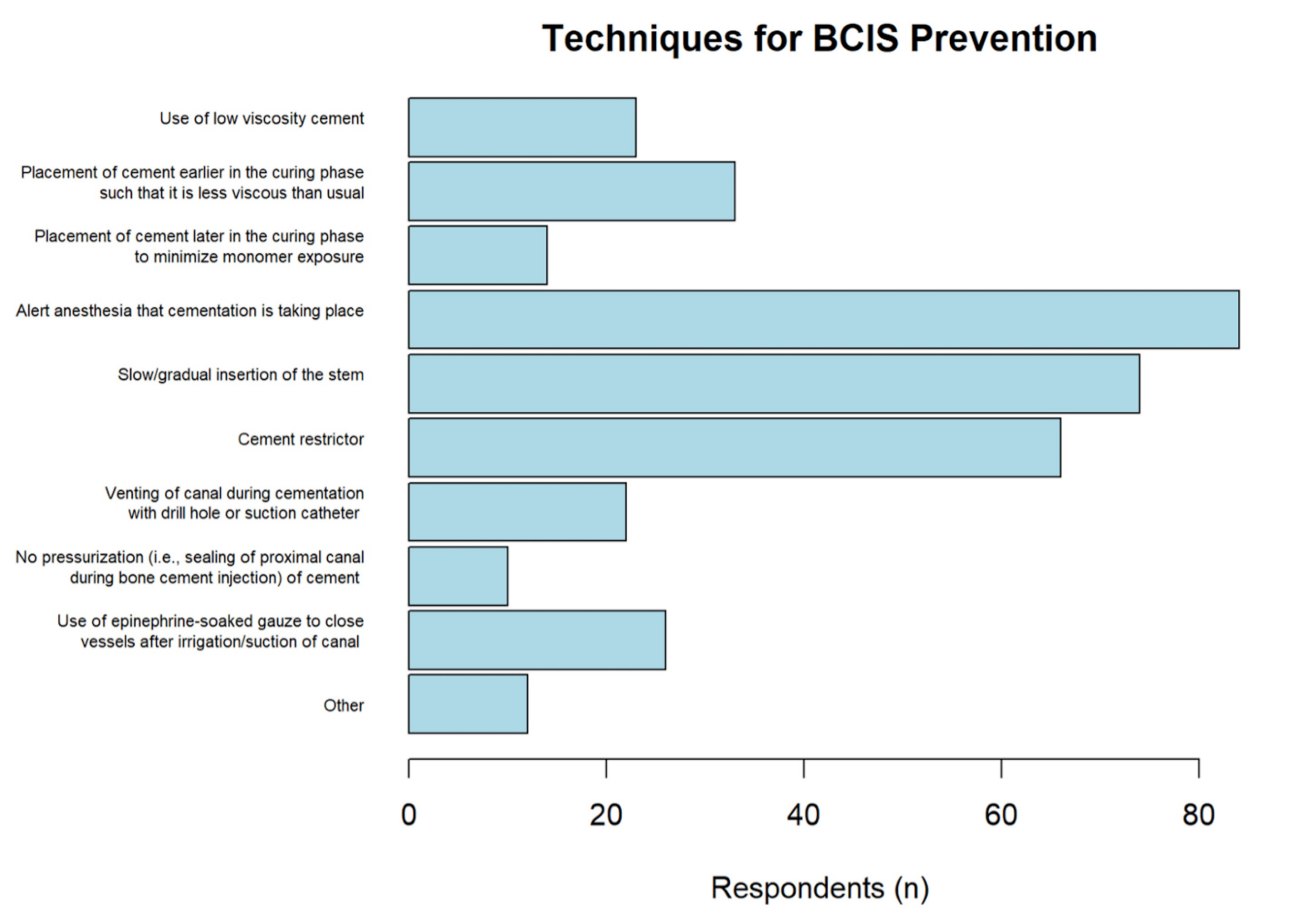
Techniques for BCIS prevention BCIS: bone cement implantation syndrome

Seventy-nine percent (n = 84) of surgeons reported using preventative strategies for BCIS. A variety of preventative strategies were employed by respondents, the most common of which included alerting anesthesia when cementing, slow/gradual insertion of the stem, and using a cement restrictor (Figure 8). Respondents who experienced severe BCIS in their patients previously were more likely to utilize preventative strategies compared with those who did not (96% vs. 78%, p = 0.00775). On average, the number of preventative techniques used per surgeon was 4.75 (SD = 1.25). 

Biomechanical Testing Results

**Figure 9:**
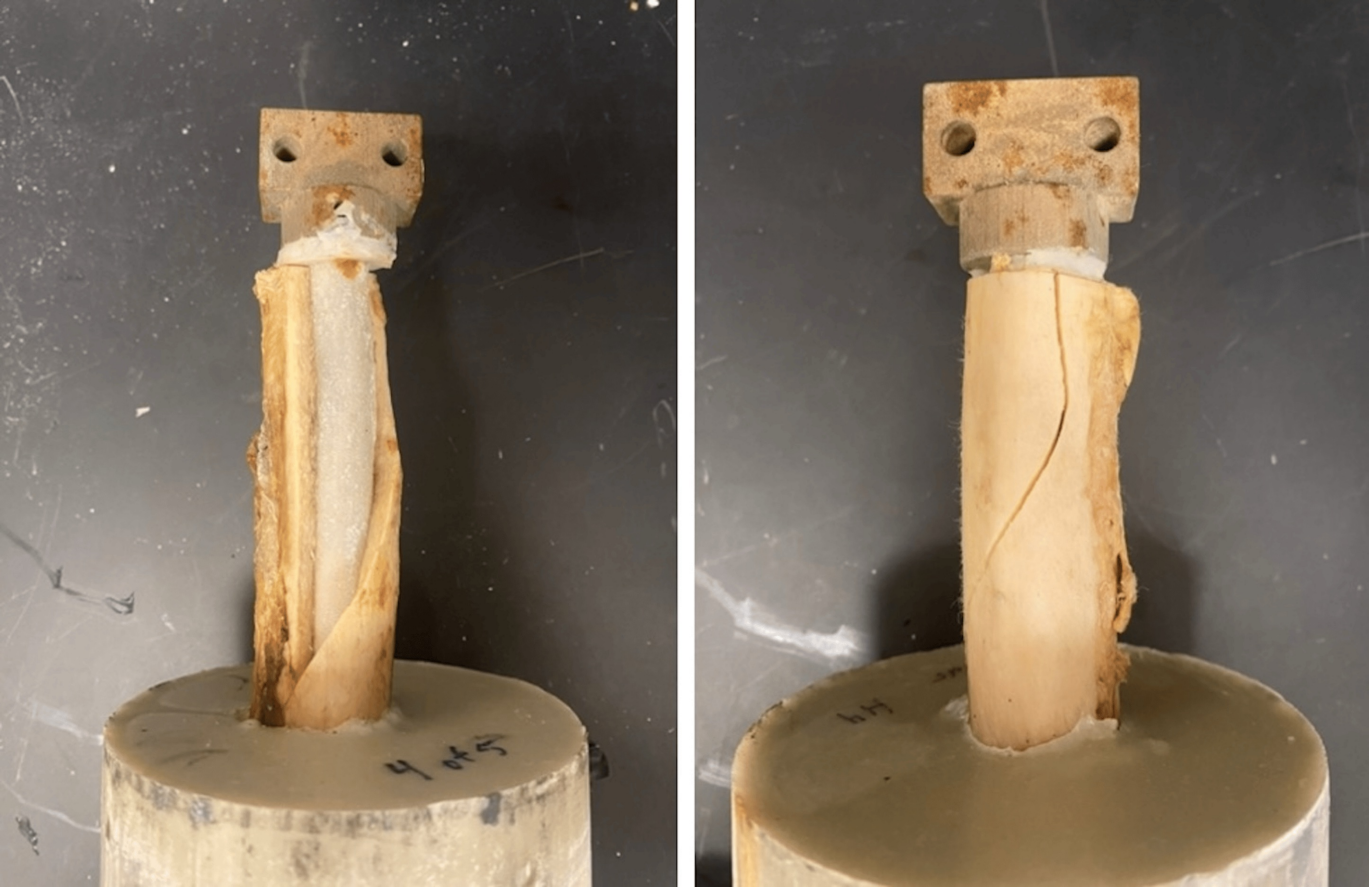
Example of failure mode with torque Example of failure mode with torque. The rectangular block with two holes at the top of the stem was designed for this study to facilitate the secure attachment of the testing machine to the stem Image credit: Vincent Y. Ng, MD; Kevin R. Aroom, MS

Finite element analysis demonstrated a 2.77% reduction in bending stiffness of the novel stem compared to a standard stem. For biomechanical testing, all of the stems, both novel and standard designs cemented into cadaveric femurs, successfully resisted 8000 N of pull-out (tensile) force. There was no significant difference in resistance to torsional force. Only one specimen (a novel stem) resisted the maximal torsion of 200 Nm. All of the other specimens failed by fracture of the cadaveric bone itself (mean peak torque to failure 140.6 Nm for the novel stem, 128 Nm for the standard stem, p = 0.62) rather than separation at the interfaces between the stem and cement or cement and bone (Figure 9). The mean difference in mean torque to failure between the stem designs in paired cadaveric femurs was 14.98 Nm. 

Implantation Pressure Testing Results

**Figure 10:**
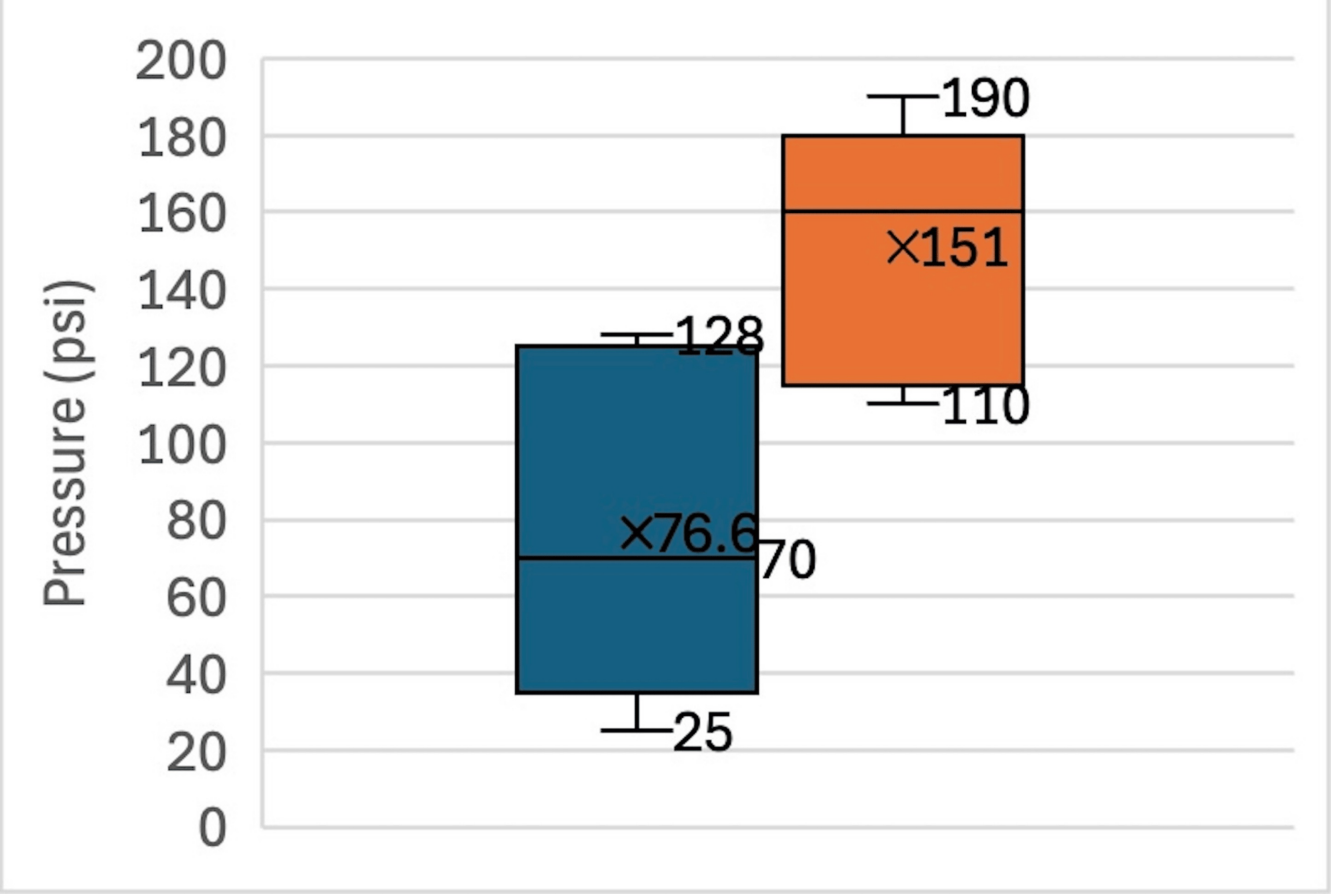
Intramedullary insertion pressure of novel vs. standard stems Blue (novel stem) and orange (standard stem) represent the range of pressures for the 10 specimens. The 95% confidence intervals are represented by brackets with median and mean values reported as well

A total of 10 stems (five novel, five standard) were successfully cemented into Sawbones, and the maximum intramedullary insertion pressures were recorded. The mean and median maximal pressures for the novel stems (76.6 psi, 70 psi) were significantly lower than for the standard stems (151 psi, 160 psi; p = 0.029) (Figure 10).

## Discussion

Cemented stems are an important component of the orthopedic surgery armamentarium, but BCIS remains a significant concern. Cemented implants are used most in elderly patients and cancer patients, two of the highest-risk groups for BCIS. This study demonstrated that many tumor surgeons have had patients with grade 3 BCIS and reported at least 1-2 mortalities in their careers due to BCIS. Most surgeons have significant concerns about BCIS, estimate the risk of BCIS to be intermediate to high in many of their patients, and take multiple measures to reduce the risk of BCIS. Nevertheless, BCIS still occurs and no significant recent advances have been made in preventing this life-threatening phenomenon. 

Existing measures to attenuate the risk of BCIS have drawbacks. The act of “alerting anesthesia” at the time of cementation to proactively prepare for impending hypotension is helpful but is only reactive, not preventative. Venting the bone near the cement plug by drilling a hole into the cortical bone will result in uncontrolled extrusion of cement into the soft tissues, often outside the exposed surgical field if a long stem is utilized. The solid stem, acting like a plunger, may simply push the cement forward and out the vented hole, resulting in limited cement actually around the implant for an adequate mantle. The hole in the cortex may act as a stress-riser for periprosthetic fracture as well. The use of low-viscosity cement can result in pressures as low as 4-9 psi (27-62 kPa) [13]. Low-viscosity cement or insertion of the stem early in the curing phase when the cement is very liquid may not result in adequate bone-cement interdigitation. According to the literature, minimally acceptable pressures of 14.5 psi (100 kPa) are needed in the laboratory to produce a stable cement mantle [15]. Very gradual insertion of the stem during cementation can limit the generation of very high intramedullary pressures, but there is a significant risk of not being able to fully seat the stem into the bone before hardening of the cement. The fact that there are surprisingly few studies focusing on the prevention of such a widespread, well-known, and potentially life-threatening condition may be attributable to the lack of significant recent advances in the field. Notably, despite the multiple intraoperative measures frequently utilized by surgeons, no major stem-related modifications are currently in use to reduce the risk of BCIS. 

This study demonstrated that a novel stem design with a longitudinal channel and an egress hole at the collar has similar bending stiffness and resistance to pull-out and torsional stress when cemented in cadaveric bone compared to a standard solid stem. The mean peak torque to failure (140 Nm) significantly exceeded the forces seen during walking and running (<40 Nm) [18]. Furthermore, when cemented into Sawbones, the novel stem generated intramedullary pressures 49% lower than the standard solid stem and yet significantly exceeded the published minimum acceptable pressures to create an adequate cement mantle. 

There are a few limitations to this study. First, long-term stability within a living subject was not evaluated. However, unlike press-fit implants, cemented stems achieve their stability through early fixation with the cement. Because the early stability of the novel stem was similar to the standard solid stems, it can be reasonably extrapolated that it would perform similarly long term. Second, the peak intramedullary insertion pressures had a relatively wide range of values when cemented into Sawbones for both the novel and standard solid stems. This variability is likely due to the numerous factors that affect insertion pressures including unavoidable variability in preparation of cement batches and speed of stem insertion. The novel stem demonstrated a reduction in insertion pressures despite this variability. Having the novel stem design as an extra safety measure against BCIS would be reassuring during actual surgery where additional uncontrolled factors are present that can introduce further variability and potential risk. 

## Conclusions

The novel stem design likely will not eliminate the risk of BCIS. Even with the longitudinal channel and egress hole at the collar, very high peak intramedullary insertion pressures can be generated if the stem is inserted late in the curing phase, with great force and speed, and into a tight canal. Nevertheless, the novel stem can reduce the pressures compared to a standard solid stem. The testing provided by this study demonstrates no significant sacrifice in stability or cement mantle strength. When combined with existing measures, the novel stem may be able to contribute to a lower risk of BCIS and intraoperative life-threatening complications for high-risk patients.

## Appendices

Appendix A. Practice experience and prevention strategies of BCIS in patients with cancer survey

Demographics 

1. Does your practice currently consist of musculoskeletal tumors at least 75% of the time?    

2. How many years have you been in practice?

Practice Experience  

3. Does bone cement implantation syndrome concern you when implanting cemented stems?

4. Have you had any cancer patients who likely experienced severe BCIS (cardiovascular collapse requiring CPR)?) 

                           a. How many would you estimate? (free text) 

                      b. How many patients would you estimate have died from intra- or postoperative complications related to this?  

5. When counseling patients preoperatively about the level of risk for a given complication, what percent do you classify as “Low,” “Intermediate,” and “High” risk?

6. In your experience, for a healthy elderly patient (>75 years) with cancer, the implantation of a cemented stem is associated with 

a. _____ (low, intermediate, high) risk of BCIS grade 1 (moderate hypoxia (SpO2 < 94%) or decrease (20%-40%) in systolic blood pressure)  

b. _____ (low, intermediate, high) risk of BCIS grade 2 (severe hypoxia (SpO2 < 88%) or decrease (>40%) in systolic blood pressure) 

c. _____ (low, intermediate, high) risk of BCIS grade 3 (cardiovascular collapse requiring CPR)  

7. In your experience, for a patient with cancer and ASA 3 or greater (substantive functional limitations; one or more moderate to severe diseases such as poorly controlled DM or HTN, COPD, morbid obesity (BMI ≥ 40), active hepatitis, alcohol dependence or abuse, implanted pacemaker, moderate reduction of ejection fraction, ESRD undergoing regularly scheduled dialysis, history (>3 months) of MI, CVA, TIA, or CAD/stents), the implantation of a cemented stem is associated with a: 

a. _____ (low, intermediate, high) risk of BCIS grade 1 (moderate hypoxia (SpO2 < 94%) or decrease (20%-40%) in systolic blood pressure)  

b. _____ (low, intermediate, high) risk of BCIS grade 2 (severe hypoxia (SpO2 < 88%) or decrease (>40%) in systolic blood pressure) 

c. _____ (low, intermediate, high) risk of BCIS grade 3 (cardiovascular collapse requiring CPR)  

8. What percent of elderly (>75) cancer patients who receive a stem (hip stem or megaprosthesis stem) do you use cement for fixation?

9. Do you use long cemented hip stems (longer than standard-length primary-type implants)?

a. What percent of the time do you use long cemented hip stems? (free text) 

b. If you do not use long cemented hip stems, is the concern for BCIS involved in your decision not to?

10. Do you use megaprosthetic cemented hip stems longer than 120 mm?

a. What percent of the time do you use megaprosthetic cemented hip stems longer than 120mm?

Current Prevention 

11. Do you employ special precautions during cementation?

a. If so, what special precautions do you use?

o Use of low viscosity cement (or early use of cement such that it is less viscous than ideal for cement mantle)  

o Alert anesthesia that cementation is taking place 

o Slow/gradual insertion of the stem 

o Cement restrictor 

o Venting of canal during cementation with drill hole or suction catheter 

o No pressurization of cement (pressurization = sealing of proximal canal during bone cement injection) 

o Use of epinephrine-soaked gauze to close vessels after irrigation/suction of canal 

o Other____ 

b. If you selected other, please elaborate.

## References

[1] Rinecker H (1980). New clinico-pathophysiological studies on the bone cement implantation syndrome. Arch Orthop Trauma Surg (1978).

[2] Donaldson AJ, Thomson HE, Harper NJ, Kenny NW (2009). Bone cement implantation syndrome. Br J Anaesth.

[3] Yang TH, Yang RS, Lin CP, Tseng TH (2021). Bone cement implantation syndrome in bone tumor surgeries: incidence, risk factors, and clinical experience. Orthop Surg.

[4] Baig MN, Curtin W, Callaghan MA, Murphy CG (2017). Catastrophic cement reaction following cementation for megaprosthesis for proximal femoral fracture. BMJ Case Rep.

[5] Garríguez-Pérez D, García-Coiradas J, Otero-Otero J, Marco-Martínez F (2020). Cement arteriovenogram after hip arthroplasty. Rev Esp Cir Ortop Traumatol (Engl Ed).

[6] Dunne NJ, Orr JF (2000). Development of a computer model to predict pressure generation around hip replacement stems. Proc Inst Mech Eng H.

[7] Zastrow RK, Rao SS, Morris CD, Levin AS (2025). The effect of anesthetic regimen on bone cement implantation syndrome in cemented hemiarthroplasty for hip fracture. J Am Acad Orthop Surg.

[8] Schwarzkopf E, Sachdev R, Flynn J, Boddapati V, Padilla RE, Prince DE (2019). Occurrence, risk factors, and outcomes of bone cement implantation syndrome after hemi and total hip arthroplasty in cancer patients. J Surg Oncol.

[9] Parvizi J, Holiday AD, Ereth MH, Lewallen DG (1999). Sudden death during primary hip arthroplasty. Clin Orthop Relat Res.

[10] Dumanlı Özcan AT, Kesimci E, Balcı CA, Kanbak O, Kaşıkara H, But A (2018). Comparison between colloid preload and coload in bone cement implantation syndrome under spinal anesthesia: a randomized controlled trial. Anesth Essays Res.

[11] Guo W, Zheng Q, Li B, Shi X, Xiang D, Wang C (2015). An experimental study to determine the role of inferior vena cava filter in preventing bone cement implantation syndrome. Iran J Radiol.

[12] Lamadé WR, Friedl W, Schmid B, Meeder PJ (1995). Bone cement implantation syndrome: a prospective randomised trial for use of antihistamine blockade. Arch Orthop Trauma Surg.

[13] Rothberg DL, Kubiak EN, Peters CL, Randall RL, Aoki SK (2013). Reducing the risk of bone cement implantation syndrome during femoral arthroplasty. Orthopedics.

[14] Pitto RP, Koessler M, Kuehle JW (1999). Comparison of fixation of the femoral component without cement and fixation with use of a bone-vacuum cementing technique for the prevention of fat embolism during total hip arthroplasty. A prospective, randomized clinical trial. J Bone Joint Surg Am.

[15] Panjabi Panjabi, MM. MM. (1983). Effect of pressurization on methylmethacrylate-bone interdigitation: an in vitro study of canine femora. J Biomech.

[16] Kapoor B, Datir SP, Davis B, Wynn-Jones CH, Maffulli N (2004). Femoral cement pressurization in hip arthroplasty: a laboratory comparison of three techniques. Acta Orthop Scand.

[17] Verdonschot N (2005). Philosophies of stem designs in cemented total hip replacement. Orthopedics.

[18] Bergmann G, Bender A, Dymke J, Duda G, Damm P (2016). Standardized loads acting in hip implants. PLoS One.

